# Thirty-Five Years of IBV Evolution in Chile Reveals a Novel Lineage and Evidence of Vaccine-Driven Recombination

**DOI:** 10.3390/v17081111

**Published:** 2025-08-13

**Authors:** Miguel Guzmán, Leandro Cádiz, Leonardo Sáenz, Héctor Hidalgo, Claudio Verdugo

**Affiliations:** 1 Laboratorio de Patología Aviaria, Departamento de Patología Animal, Facultad de Ciencias Veterinarias y Pecuarias, Universidad de Chile, Santiago 8820808, Chile; miguelguzman@uchile.cl (M.G.);; 2 Núcleo de Investigación en One Health (NIOH), Facultad de Medicina Veterinaria y Agronomía, Universidad de las Américas, Campus Maipú, 5 de Abril 620, Santiago 9250000, Chile; 3 Facultad de Ciencias Veterinarias y Pecuarias, Universidad de Chile, Santiago 8820808, Chile; 4 Instituto de Patología Animal, Universidad Austral de Chile, Campus Isla Teja, Edificio BID C 3° Piso, Valdivia 5090000, Chile; 5 Center for Surveillance and Evolution of Infectious Diseases, Universidad Austral de Chile, Valdivia 5090000, Chile

**Keywords:** infectious bronchitis virus, viral phylodynamic, poultry

## Abstract

Infectious bronchitis virus (IBV) remains a major threat to poultry health worldwide due to frequent genetic changes mainly driven by recombination and limited cross-protection between genotypes. In this study, we analyzed IBV strains collected from clinical outbreaks in Chile between 1986 and 2021 to assess the long-term impacts of live-attenuated vaccines (Massachusetts and 4/91) on viral evolution. Phylogenetic analysis of the S1 and N genes revealed four major lineages circulating in Chile—GI-1, GI-13, GI-16, and a novel monophyletic clade we propose as GI-31. The latter, identified in isolates from 1986 to 1988, is highly divergent (22–24%) from other known lineages, representing a previously unreported South American IBV variant. Despite widespread Mass vaccination, genetically distinct field strains circulated during the 1980s, facilitating potential recombination with GI-1 vaccine-derived strains, including evidence of shared ancestry with GI-11, an endemic lineage from Brazil. Non-recombinant GI-16, likely introduced from Asia, was detected in isolates from 2009. Notably, a recombinant strain emerged in 2015, four years after 4/91 vaccine introduction, indicating vaccine–field-strain genetic exchange. By 2017, isolates with >99% identity to the 4/91 strain were recovered, suggesting vaccine-derived variants. In 2021, GI-1 re-emerged, showing recombination signatures between GI-1 and GI-13 (4/91-derived) strains, likely reflecting suboptimal or inconsistent vaccination strategies. Selection analyses showed strong purifying selection across most of the S1 gene, with limited sites under positive selection in the receptor-binding domain. Phylodynamic reconstruction revealed time-structured evolution and multiple introduction events over 35 years, with lineage-specific tMRCA estimates. Collectively, these findings highlight the emergence of a novel lineage in South America and demonstrate that vaccine use, while mitigating disease, has significantly shaped the evolution of IBV in Chile. Our results underscore the importance of continuous genomic surveillance to inform vaccine strategies and limit recombinant emergence.

## 1. Introduction

Infectious bronchitis (IB) remains a major infectious disease affecting the global poultry industry, caused by the avian infectious bronchitis virus (IBV), a positive-sense, single-stranded RNA virus classified within the genus *Gammacoronavirus* (Fam. *Coronaviridae*) [1]. The virus exhibits primary tropism for the upper respiratory epithelium, where initial replication leads to acute respiratory signs, including nasal discharge, sneezing, and conjunctivitis [2]. Transient viremia may facilitate dissemination to secondary target organs, such as the kidneys and oviduct, depending on the viral strain, resulting in nephritis, fluid imbalance, urate deposition, and impaired reproductive performance characterized by reduced egg production and quality [3,4]. Although flock-level IBV infection rates can be as high as 100%, mortality is typically strain-dependent and often exacerbated by concurrent secondary infections [5]. The control of IB primarily relies on the strategic use of both live-attenuated and inactivated vaccines [6], with a variety of homologous and heterologous IBV vaccines currently available. Despite widespread vaccination efforts, effective control remains challenging owing to the intrinsic genetic plasticity of IBV. Like other coronaviruses, IBV has an intrinsically high mutation rate [7] and is prone to RNA–RNA recombination events [8], facilitating rapid viral evolution. The frequent occurrence of these drivers of evolutionary change in coronaviruses allows for the rapid exchange of beneficial mutations, promoting the continual emergence of antigenically novel IBV variants worldwide [9,10,11,12] and contributing to vaccine failure, particularly in high-density commercial poultry systems [13].

Of particular concern is the occurrence of homologous recombination between vaccine-derived and wild-type IBV strains, which has been reported globally, resulting in the emergence of new serotypes [14,15,16,17,18,19] with altered tissue tropism [12,20] or enhanced virulence [21]. While these processes can occur throughout the ~27.6 kb IBV genome, they are particularly frequent in the gene encoding the spike (S) glycoprotein [22]. The S protein, specifically its S1 subunit, plays a critical role in host cell receptor binding and the induction in virus-specific neutralizing antibodies [23], making it a key determinant of viral antigenicity and immune escape [3,9,24,25,26,27,28,29]. Due to its high variability, the S1 subunit serves as a useful marker for the study of the evolutionary dynamics of viral populations. Based on S1 gene sequences, IBV is currently classified into ten major genotypes (G-I to G-VI [30], GVII-1 [31], GVIII-1 and GVIII-2, and GIX-1 [32,33]). G-I represents a highly diverse genotype encompassing at least 31 lineages (GI-1 to 27 [30], GI-28 [34], GI-29 [35], GI-30.1 [36], and GI-30.2 [32]).

In South America, ongoing surveillance has identified the persistent circulation of at least two IBV lineages in commercial poultry farms [32]. The endemic lineage GI-11 (also known as South America I or SAI) has been documented in Brazil, Uruguay, Argentina, and Colombia [37,38,39], whereas the globally disseminated GI-16 lineage (also referred to as Q1 or Asia/South America II, A/SAII) has been reported across most countries in the region [37,40,41,42,43]. Additionally, lineage GI-1, associated with Massachusetts (Mass) vaccine strains, has been detected in field samples from Argentina, Brazil, and Colombia, indicating possible reversion to virulence or the circulation of vaccine-derived viruses [44,45]. More recently, the emergence of lineage GI-23 in Brazil has raised concerns, as it has been detected in multiple commercial broiler flocks, suggesting its successful establishment and spread [46]. In Chile, diverse lineages, including GI-1 [47], GI-13 [48], and GI-16 [42], have been implicated in field outbreaks, highlighting the increasing genetic complexity and geographic distribution of IBV in the region.

Poultry farming is one of the most important livestock sectors in Chile, with an estimated annual growth rate of 3% and a total output exceeding 700,000 tons by 2022 [49]. IBV was first reported in the country in 1967 [50] and was later isolated from a 6-week-old broiler flock in 1975, with antigenic characterization revealing a close relationship to the Mass serotype [47]. These early findings have led to the adoption of a live-attenuated Mass-type vaccine as the cornerstone of national IBV control strategies for several decades. Nevertheless, other antigenically distinct serotypes, including Connecticut-like strains, were also identified co-circulating in Chilean poultry populations [47,51,52]. Currently, IBV is recognized as endemic in Chile and is classified as a notifiable disease under national veterinary health regulations [53]. For many years, it was presumed that GI-1 was the sole lineage circulating in the country, which contributed to a lack of sustained molecular surveillance. However, this assumption was challenged in 2008 with the detection of the GI-16 (Q1) lineage isolated from several Mass-vaccinated flocks experiencing clinical outbreaks [42]. In response, the 4/91 vaccine strain was introduced in 2011 to complement the existing Mass-based immunization strategy [54,55], leading to partial control of major outbreaks caused by circulating strains [56]. Our previous study identified and antigenically characterized field isolates of GI-13 and GI-16 circulating between 2009 and 2017 [48]. In the present study, we expand upon this analysis by examining IBV samples collected over a 35-year period (1986 to 2021) to elucidate the temporal dynamics of viral genetic diversity in Chile and to assess the impact of live-attenuated vaccine introduction on the evolution of circulating IBV populations in Chile.

## 2. Materials and Methods

### 2.1. Virus Isolates

Seventeen field IBV isolates were obtained from clinical outbreaks that occurred between 1986 and 2021 across 12 commercial poultry farms located in Central Chile (Table 1). Four isolates were collected in 1986 and 1988 from broilers and layers exhibiting respiratory symptoms, egg production drop, and/or nephritis on farms located in the Valparaíso and Metropolitana regions. These samples were inoculated into embryonated chicken eggs (ECEs), and the virus-containing allantoic fluid was harvested and subsequently lyophilized for long-term storage, as previously described [51]. Four viruses collected in 1986 and 1988 were lyophilized after isolation in ECE. The remaining isolates were obtained in 2009, 2015, 2017, and 2021 from commercial broiler and layer flocks located in the Valparaíso, Metropolitana, O’Higgins, and Maule regions. All flocks had been vaccinated with the Mass (GI-1) live attenuated vaccine; those sampled in 2015, 2017, and 2021 had also received the 4/91 (GI-13) vaccine strain.

Tracheal swabs were suspended in tryptose phosphate broth (TPB), while kidney tissues were subjected to freeze–thaw cycles and centrifuged, and the resulting supernatants were collected. All samples were inoculated into the allantoic cavity of 10-day-old SPF embryonated eggs pre-treated with streptomycin and penicillin (1:10 dilution). After three to five passages, allantoic fluids were harvested 48 h post-inoculation and stored at −80 °C for downstream analysis.

### 2.2. RT-PCR and Sequencing

Viral RNA was extracted from allantoic fluids using the PureLink™ viral RNA/DNA Mini Kit (Invitrogen, Waltham, MA, USA) following the manufacturer’s protocol. The complete S1 subunit gene (~1600 bp) and a partial fragment of the nucleocapsid (N) gene (~1000 bp) were amplified as previously described [57,58] with the following primers: IBVS1-F: 5-AACTCTGCACGCAAATTA-3, IBVS1-R: 5-TGTTGTCGCAAACAGGACC-3; and IBVN2-F: 5′-CTGGAAAAACAGACGCCCCA-3′, IBVN2-R: 5′-CTCCAAGTGCTGAATCGCCC-3′, respectively. An RT-PCR was performed using the SuperScript™ III One-Step RT-PCR System with Platinum™ Taq High Fidelity DNA Polymerase (Invitrogen, USA) with a high-fidelity DNA polymerase under the following thermal profile: 30 min at 55 °C, 2 min at 94 °C, followed by 40 cycles of 15 s at 94 °C, 30 s at 49 °C (S1) or 48.4 °C (N), and 2 min at 68 °C, with a final extension at 68 °C for 5 min. Ultrapure water and a commercial vaccine were included as negative and positive controls, respectively. PCR amplicons were purified using the PureLink^®^ Quick Gel Extraction Kit (Invitrogen, Waltham, MA, USA), following the manufacturer’s instructions. Purified products were then quantified and sequenced bidirectionally using an ABI 3730XL Genetic Analyzer.

### 2.3. Phylogenetic and Selection Analysis

Raw sequence reads were edited, assembled, and submitted to GenBank (Table 1). Genotypes and lineages were assigned based on the phylogenetic inference of a curated dataset of 139 S1 gene sequences (1618 bp; Supplementary Table S4) using the lineage classification proposed by Valastro et al. [30]. This dataset included representative sequences from all currently recognized IBV genotypes and lineages. Sequence alignments were performed with MAFFT v7.2 [59], and the best-fit nucleotide substitution model (GTR + I + G) was identified using jModelTest v2.1.7 [60]. Phylogenetic trees were inferred using the maximum-likelihood (ML) method implemented in PhyML v3.0 [61], with node support assessed via transfer bootstrap expectation (TBE) based on 1000 replicates [62]. The final trees were visualized using FigTree v1.4.2 [63]. A genetic distance matrix for both nucleotide and amino acid S1 gene sequences was generated in MEGA X [64] to evaluate the divergence between Chilean clades and reference lineages. To assess potential recombination events and tree topology congruence, a dataset of 54 partial N gene sequences (1000 bp) retrieved from GenBank was aligned and analyzed in parallel.

Selection pressures on the S1 gene were evaluated using the ratio of non-synonymous to synonymous substitutions per site (dN/dS) using the HyPhy package implemented in the Datamonkey web server [65]. For this purpose, a dataset including 504 S1 gene sequences from different years and countries was retrieved from GenBank, including Chilean samples. Codon-level selection analyses were conducted using the single likelihood ancestor counting (SLAC); fixed effect likelihood (FEL); and fast, unconstrained Bayesian approximation (FUBAR) methods [66,67,68].

To estimate evolutionary rates and the time to the most recent common ancestor (tMRCA), a Bayesian Markov chain Monte Carlo (MCMC) analysis was performed using the BEAST2 package [63] with a dataset of 349 complete S1 sequences (1620 bp) spanning 26 countries and collected between 1954 and 2021. Temporal signals and clock-likeness of the data were evaluated using TempEst v1.5 [69] by regression of root-to-tip genetic distances against sampling dates. Molecular clock models, including strict and uncorrelated lognormal relaxed clocks, were tested alongside a coalescent Bayesian Skyline tree prior. Three simultaneous MCMC chains were run until convergence, and the outputs were combined using LogCombiner [63]. Phylodynamic analysis supported the use of an uncorrelated lognormal relaxed molecular clock over a strict clock model, as indicated by higher marginal likelihood estimates using both path sampling (PS) and stepping-stone (SS) methods [70] (PS = −75,646; SS = −75,699 vs. PS = −76,538; SS = −76,580). The final maximum clade credibility tree was summarized using TreeAnnotator and visualized in FigTree v1.4.2 [63].

## 3. Results and Discussion

**Figure 1:**
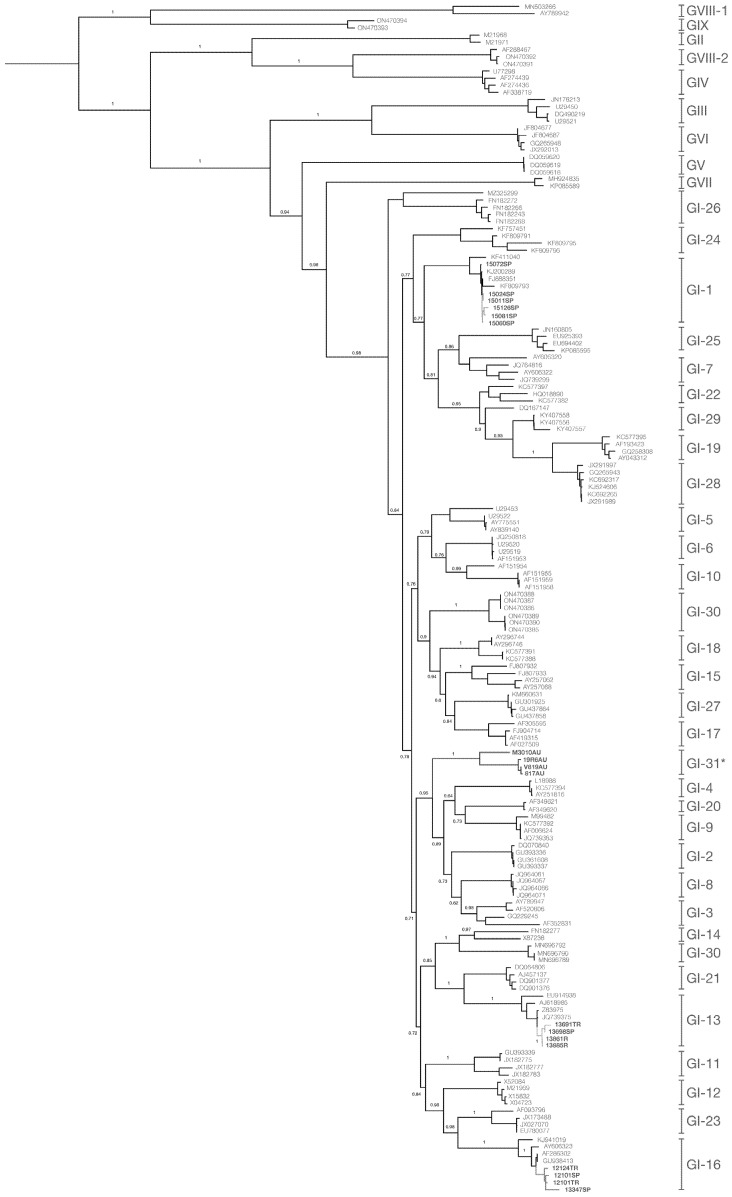
Maximum-likelihood phylogenetic tree of the S1 gene sequences of IBV following Valastro et al.’s classification [30]. Chilean sequences from this study are colored blue (samples from 1986 to 1988), red (samples from 2009 and 2015), green (samples from 2017), and yellow (samples from 2021). Vaccine strains are located within GI-1 (Massachusetts-type vaccines) and GI-13 (793/B-type vaccines). * denotes the herein-proposed lineage GI-31. Sequences from GVIII-1 and GIX-1 were used as a root. Numbers indicate bootstrap branch supports.

Phylogenetic analysis of the S1 gene revealed that all Chilean sequences clustered within G-I, following the classification proposed by Valastro et al. [30] (Figure 1). Four distinct monophyletic lineages were identified circulating in Chile since 1986: GI-1, GI-13, GI-16, and a novel clade that has not been previously described. Temporal clustering was evident in most lineages, indicating time-structured evolutionary dynamics and defined periods of emergence and circulation.

### 3.1. Identification of a Novel Chilean Lineage

The S1 gene sequences obtained from samples collected between 1986 and 1988 formed a unique monophyletic group, with no related sequences previously reported. The nucleotide and amino acid intra-clade genetic diversities were 6.88% and 6.83%, respectively (Supplementary Materials Tables S1 and S2). This clade diverged by 22.76 to 25.58% from related lineages GI-2, GI-3, GI-4, GI-8, GI-9, and GI-20 (Supplementary Materials Table S3), supporting its classification as a novel lineage, which we propose to name GI-31. Recently, Rafique et al. [71] referenced a virus isolate labeled as GI-31, originally reported by Bali et al. [72], based on a single sequence identified in Côte d’Ivoire (D2334/11/2/13/CI; Accession: OM691146). However, according to the lineage designation criteria established by Valastro et al. [30], the classification of a novel IBV lineage requires a minimum of three non-identical S1 gene sequences, forming a distinct phylogenetic cluster and harboring adequate nucleotide divergence from established lineages. As the proposed African GI-31 lineage is supported by only a single sequence and lacks additional genetic, epidemiological data, it should be regarded as an unclassified variant rather than a confirmed, recognized lineage. In contrast, our study provides robust support for a novel lineage based on four independent and temporally linked isolates collected between 1986 and 1988 from commercial poultry farms in Central Chile. These sequences form a well-supported monophyletic clade, exhibiting intra-lineage nucleotide and amino acid divergence exceeding 6% and are clearly distinct from all previously described GI lineages in S1 phylogenies. Therefore, we propose to assign the designation GI-31 to this novel Chilean lineage, which fulfills all established criteria [30].

**Figure 2:**
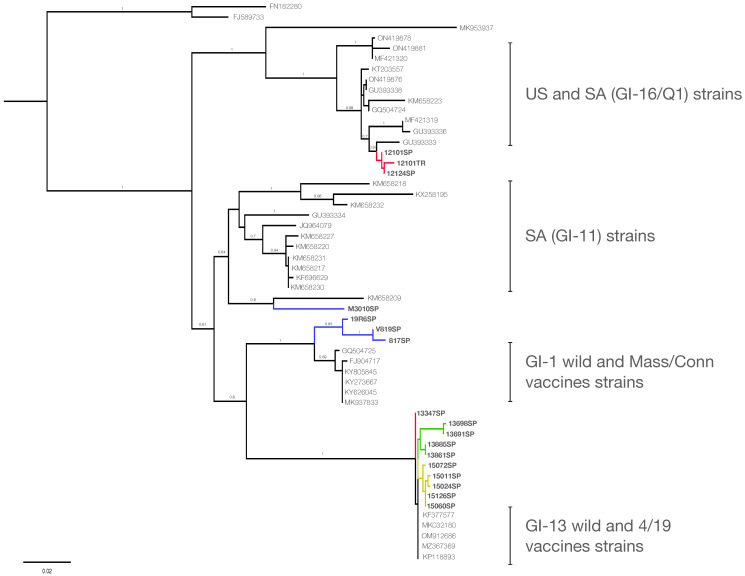
Maximum-likelihood phylogenetic tree of the N sequences of IBV. Chilean sequences from this study are colored blue (samples from 1986 to 1988), red (samples from 2009 and 2015), green (samples from 2017), and yellow (samples from 2021). Numbers indicate bootstrap branch supports.

The ancestral lineages GI-2, GI-3, and GI-4 are associated with isolates from the USA collected in the 1950s and 1960s and later detected in Asia [30]. Lineages GI-8 and GI-9 correspond to endemic strains from the USA, whereas GI-20 is a lineage of viruses isolated exclusively from Canada [30]. Chilean samples belonging to the GI-31 clade were obtained from Mass-vaccinated commercial farms in Central Chile experiencing IBV outbreaks in the late 1980s [51]. These isolates were demonstrated to be highly virulent in experimentally inoculated chickens and serologically distinct from known IBV serotypes, including Mass (GI-1), Holte (GI-2/GI-4), and Ark (GI-9) [51]. Currently, there is no evidence to support the continued circulation of this variant, which may now be extinct or potentially persist at low frequency in remote or under-surveyed locations. Further surveillance is necessary to improve our understanding of the origin, spread, and epidemiological status of this clade, as previously emerging or undetected lineages have been identified [73]. Phylogenetic inference from the N gene (Figure 2) revealed that most viruses isolated in the 1980s were grouped into a clade with sequences from the GI-1 lineage, the earliest IBV lineage described in Chile in 1975 [46]. Despite vaccination with the Mass serotype [51], genetically divergent IBV strains were circulating, representing an ideal scenario of recombination opportunities between vaccine and field strains. Further genomic approaches would allow us to map and identify specific recombination breakpoints along the viral genome and assess the role of Mass vaccines in shaping the evolution of local IBV strains. Notably, the N gene of one isolate (M3010SP) shared a common ancestor with viruses belonging to the GI-11 strain, an indigenous South American lineage endemic to Brazil but also reported in Argentina, Uruguay, and Colombia [37,38,39]. Although only recently sequenced from samples collected in the 2000s, GI-11 likely emerged between the 1950s and 1960s [37,74]. The detection of signatures of GI-11-like sequences in Chile suggests the potential circulation during this temporal gap, between the 1960s and 2000s, characterized by limited molecular surveillance [38,39]. GI-11 strains have been shown to be antigenically different from the Mass serotype [75], suggesting limited vaccine efficacy and increasing the likelihood of recombination with local strains. GI-11 has been shown to recombine extensively with the widespread GI-16 in other South American countries [76], and similar interactions in Chile may have contributed to the emergence of novel recombinant variants. Hence, the Mass vaccine introduced to Chile was likely unable to protect individuals infected with these field variants, allowing the recombination of GI-11 in Chile. However, S1 sequences of GI-11 have not been detected in Chile, implying localized circulation restricted to a few geographical areas, at a very low frequency, and/or with low virulence, requiring further surveillance efforts to determine the presence of this variant.

### 3.2. Emergence and Persistence of GI-16 in Chile

S1 gene sequences obtained from samples collected in 2009 and 2015 formed a monophyletic clade within the GI-16 lineage (Figure 1), with an intra-clade diversity of 2.3% (nucleotide) and 3.9% (amino acid), and a 5% divergence from global GI-16 sequences (Supplementary Materials Tables S1–S3). These Chilean sequences are thus consistent with the GI-16 lineage, which includes the Q1 strain, a widespread genotype associated with severe nephropathogenic disease and significant economic losses. This strain was initially identified in Italy in 1993, before spreading to China, the Middle East, and South America [73], including Brazil, Argentina, and Uruguay in 2009 [77] and Peru in 2014 [78]. The introduction of G1-16 to South America was likely from China and/or Taiwan, followed by local spread among neighboring countries [73]. Consequently, the phylogenetic proximity of Chilean isolates to Chinese Q1 strains supports their introduction from Asia, followed by regional dissemination. In Chile, GI-16 isolate was first identified in samples collected in October 2008 from outbreaks characterized by respiratory and renal symptoms, poor growth, and reduced egg production [42]. Because the Mass-793B protectotype provided effective immunity against GI-16 [42], the 4/91 vaccine (GI-13) was officially introduced in 2011 as part of a combined Mass-4/91 vaccination strategy against field strains [56]. Although this combination improved protection against antigenically different strains, the immunity level and efficacy reached by heterologous strains vary significantly depending on the genetic distance from circulating viruses [79,80]. The non-sterilizing immunity elicited by IBV allows viral replication and continued viral evolution in partially protected chickens, potentially leading to vaccine escape or recombinant emergence. This was evidenced by the N gene phylogeny (Figure 2), where the sequences from samples of 2009 (12101TR-09, 12124SP-09, and 12101SP-09) clustered with sequences from the USA and South America of the GI-16 lineage, similar to the S1 sequence phylogeny, suggesting the circulation of non-recombinant GI-16 before the arrival of the 4/91 vaccine. However, the isolate from 2015 (13347SP-15), sampled four years after the 4/91 vaccine’s introduction, clustered with 7/93 virus sequences and had 99% identity with the 4/91 vaccine in the N gene, despite being grouped with GI-16 based on the S1 sequences. Such topological discrepancies between gene trees suggest the occurrence of recombination between field G1-16 strains and the vaccine 4/91 strains, a phenomenon frequently documented in viral genomes, including coronaviruses and other RNA viruses [81,82,83,84,85]. While direct identification of recombination breakpoints, parental strains, or genomic regions was not performed, this incongruence provides compelling indirect evidence of recombination [8,86,87]. Future research using full-genome sequencing is necessary to detect recombination junctions and conclusively define the genetic architecture of these recombinant viruses. Similar events of recombination between the 4/91 vaccine and IBV field strains have been reported globally, including China [88,89], Russia [90], Egypt [14], and Mexico [85], and even with more than one vaccine as a parental strain [19,91,92]. Notably, the Chilean case showed a four-year interval between vaccine introduction and the first recombinant detection, suggesting a temporal lag in recombination emergence. This may serve as a guideline for other countries planning heterologous vaccine use, highlighting the importance of genomic surveillance during and after vaccine introduction.

### 3.3. Emergence of GI-13 Strain

The Chilean sequences from viruses isolated in 2017 clustered together and shared ancestry with the sequences of the GI-13 lineage, showing a divergence of 0.9% from other GI-13 strains (Supplementary Materials Table S3) and only 0.7% genetic distance from the 4/91 vaccine. The GI-13 lineage includes vaccines and field strains, such as the 793/B, 4/91, and CR88 strains [30]. The demonstrated effectiveness of the protectotype of 4/91 with the Mass vaccine against heterologous strains allowed the introduction of the 4/91 vaccine in many countries where the strain was absent, including Chile in 2011 [42,54]. However, after the introduction of the 4/91 vaccine, GI-13 has since emerged as a recombinant major parent in virulent strains with wild-type strains in several countries across Central and South America [14,19,93,94]. The isolate 13347SP-15 from 2015 exemplifies such an event, representing an early recombinant. Furthermore, by 2017, evidence suggests a transition from recombinant strains GI-16 with GI-13 toward GI-13 vaccine-derived variants. These isolates showed a high genetic identity with the 4/91 vaccine strain (KF377577). In this context, a critical challenge is distinguishing vaccine-derived or field-adapted strains from the original vaccine virus. This is particularly difficult with the 4/91 strain, as attenuated vaccines and virulent field strains often share identical S1 gene sequences, making discrimination based only on S1 phylogeny unreliable [89]. Nevertheless, several lines of evidence allow us to infer that the 4/91-like viruses identified in 2017 were not residual vaccine viruses but vaccine-derived or recombinant variants. All affected flocks had been vaccinated at least four weeks prior to sampling—and in some cases up to 23 weeks earlier—well beyond the known window of vaccine virus shedding, which typically lasts 5–7 days and rarely exceeds 14 days post-vaccination [79]. Second, the viruses were isolated from clinically affected birds, which is inconsistent with the expected behavior of attenuated vaccine strains. Third, phylogenetic analysis of the N gene from isolate 13347SP-15 showed clustering with 4/91-like sequences despite grouping with GI-16 in the S1 gene tree, a phylogenetic incongruence that suggests signatures of recombination between field and vaccine strains. These data strongly support that the viruses circulating in 2017 represent either vaccine-derived escape variants or recombinants that evolved under field conditions. Similar recombination events involving the 4/91 strain have been reported globally, including in China, Egypt, and Mexico [14,84], underscoring the importance of genomic surveillance following the introduction and widespread use of live-attenuated vaccines.

### 3.4. GI-1 Re-Emergence and Recombination in 2021 Outbreaks

All S1 gene sequences from samples collected in 2021 clustered into a single group within the GI-1 lineage, exhibiting a low intra-clade diversity (0.85% nucleotide; 1.6% amino acid) and a 1.6% divergence from reference GI-1 sequences (Supplementary Materials Tables S1–S3). These findings suggest the re-emergence of strains derived from live attenuated Mass-type vaccines used in Chile, although some genetic drifting has occurred, as inferred from the distance observed. GI-1 is one of the most genetically stable and widespread IBV lineages worldwide [30] and has been historically present in Chile since the first isolates were described in the 1960s [47]. The re-emergence of GI-1 in Chile, causing clinical outbreaks, may reflect inconsistencies and heterogeneities in field vaccination strategies applied to control strains derived from GI-13 and GI-16 lineages using 4/91 and Mass vaccines. For instance, the use of both vaccines together, alternating vaccines, or the use at different proportions along the schedule may have allowed the persistence and escape of Mass-derived mutants that can cause clinical symptoms [2,95,96]. In contrast, the analysis of the N gene sequences revealed clear phylogenetic incongruences compared with S1 gene phylogeny. All sequences from samples isolated in 2021 clustered with sequences of the 793/B group, showing 99% nucleotide identity with the 4/91 vaccine strain and diverging from their GI-1 S1-based classification [30]. The switch of sequences across the tree and the genetic identity suggest that the sequences from 2021 are recombinants, most likely between the GI-1 (Mass-derived) and GI-13 (4/91-derived) strains [90,91]. Recombination between the 4/91 vaccine and different IBV lineages, such as GI-19 and GI-28, has been previously reported in China [91,92]. Although direct mapping of recombination breakpoints was not determined, the observed gene tree discordance between the S1 and N genes is consistent with the disruption of phylogenetic congruity across genomic regions by recombination events [8,86,87]. To rigorously confirm these recombination events and precisely locate breakpoints, whole-genome sequencing and detailed mapping of recombination-prone regions will be required.

### 3.5. Selection Pressure on the S1 Gene

Across all lineages, the mean dN/dS ratio observed in the 530 codons of S1 gene was 0.66, indicating predominant purifying selection. For Chilean clades, ratios were even lower at 0.03 (1986–1988), 0.015 (2009–2015), 0.013 (2017), and 0.01 (2021), suggesting the strong evolutionary constraint of variants containing non-functional mutations in the S1 gene or changes involving traits against the viral fitness, likely driven by the functional importance of the spike protein in receptor binding [97,98]. However, three codons (N34, T49, and V67) were under significant positive selection (dn/ds ratio > 1). These codons were located in the hypervariable region 1 (HVR-1) of the S1 gene [99], which is part of the receptor-binding domain (RBD) in the N-terminal domain of the spike protein and is known to be critical for cellular host attachment [100] and immunological evasion [19,101]. The S gene encodes for the spike protein, a surface protein that mediates the receptor binding and membrane fusion, promoting viral entry into the host cells [102]. Thus, positive selection is driving sequence evolution in limited sites at the N-terminal domain of the S1 gene associated with the receptor binding, whereas a more stringent purifying selection constrains the protein evolution in (most of) the remaining gene sequence.

### 3.6. Phylodynamic Analyses

**Figure 3:**
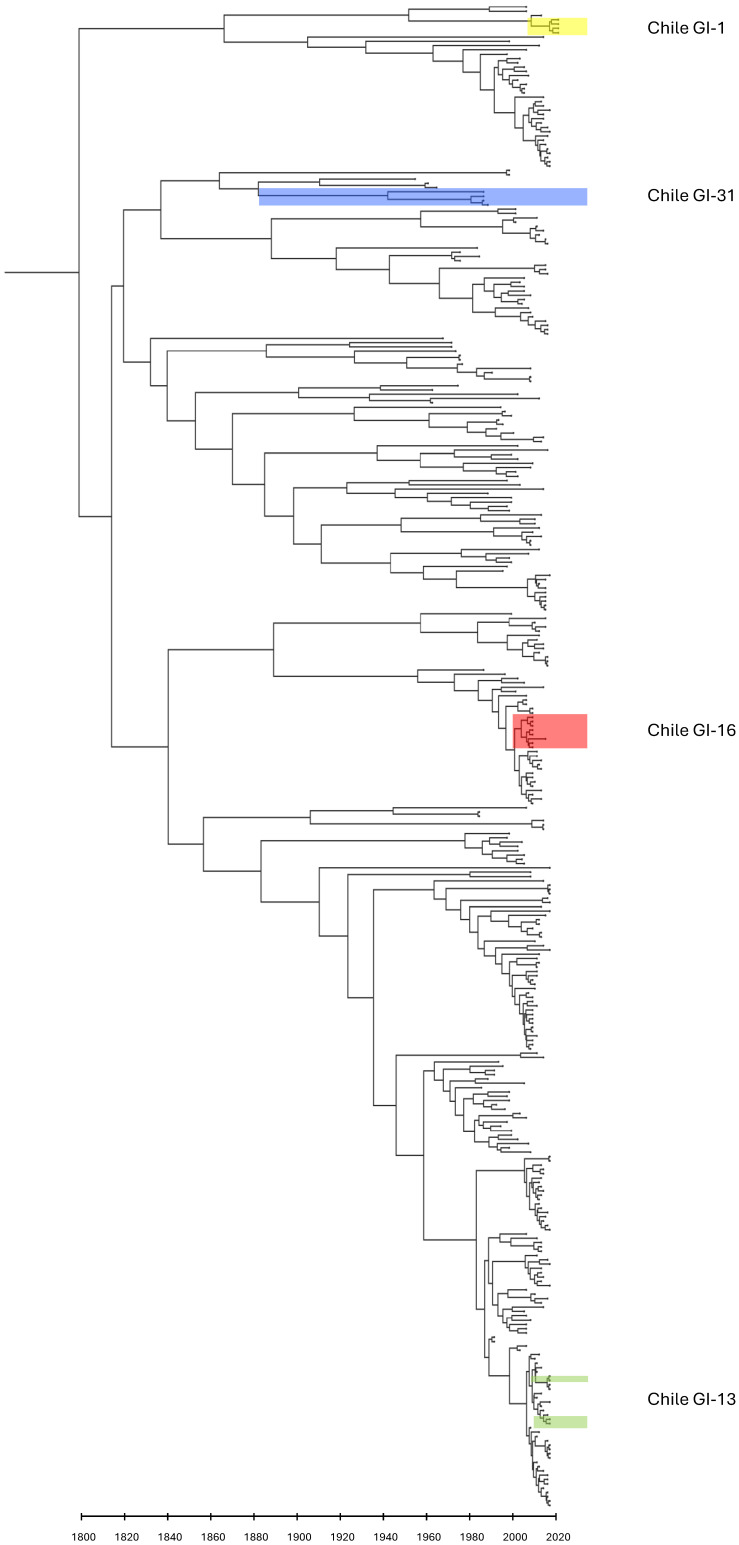
Maximum clade credibility tree of the S1 gene from global lineages of IBV. Chilean sequences from this study are colored blue (samples from 1986 and 1988), red (samples from 2009 and 2015), green (samples from 2017), and yellow (samples from 2021).

Using a dataset of S1 gene sequences from global IBV strains collected between 1954 and 2021, the estimated time to the most recent common ancestor (tMRCA) was approximately 224 years before the most recent sample (95% highest posterior density [HPD]: 166–300 years), suggesting that the observed genetic diversity likely originated around the early 1800s (Figure 3). However, this estimate should be interpreted cautiously due to the limited availability of historical sequences from the 1950s and 1960s, which increases uncertainty in dating the root node. Although IBV was first isolated in the 1930s [103], the placement of the root in the early 1800s remains plausible, given the deep genetic divergence among currently circulating strains. The time-scaled Bayesian phylogeny grouped Chilean sequences into four distinct clades, consistent with their sampling dates. The tMRCA for the Chilean GI-31 lineage (1986–1988) was estimated at 1881 (95% HPD: 1829–1923), diverging from a clade including U.S. strains associated with the Holte serotype (GI-2/GI-4), as well as the Gray and JMK serotypes (GI-3), which emerged in the 1950s and 1960s. This pattern aligns with the topology observed in maximum-likelihood phylogenetic trees of the S1 gene. An internal node representing the divergence of the Chilean isolate M3010AU was dated to approximately 1941, indicating a ~60-year gap in sequence data from divergence to the earliest sampled U.S. relatives. This gap underscores the need for historical sequence data to better resolve the origin of the GI-31 lineage. Similar deep divergence times have been reported for other lineages, such as GI-7, estimated to have originated around 1886 [104], GI-16 in 1905 [73], and GI-23, dated to 1933 [105]. IBV was first described in South America, affecting commercial poultry farms from Brazil in the 1950s [106]. These findings suggest that earlier field strains were already circulating in South America, supporting the hypothesis that ancestral strains remain unsampled or have become extinct, yet played a significant role in shaping the current global diversity of IBV. Sequences from the Chilean clade of the GI-16 lineage collected between 2009 and 2015 showed an estimated tMRCA of 2001 (95% HPD: 1997–2004). These strains diverged from a clade containing sequences from Argentina and Uruguay, supporting the hypothesis that Chinese Q1-type strains were introduced into multiple South American countries in the early 2000s and subsequently spread locally within Chile. This introduction timeline and regional dissemination pattern align with findings reported by Franzo et al. [73]. Sequences from isolates obtained in 2017 formed two distinct clades with similar tMRCA estimates: one dated to 2015 (95% HPD: 2012–2016) and the other to 2016 (95% HPD: 2014–2017). These closely related clades are consistent with the introduction of the 4/91 live-attenuated vaccine in Chilean poultry in 2011, which, together with the phylogenetic position observed in the ML tree of the S1 and N gene and the 4/91 strain derived from live attenuated vaccines, are the origin of the observed circulating isolates, escapes that occurred on multiple opportunities. The most recent Chilean isolates, obtained in 2021 and belonging to the GI-1 lineage, exhibited a tMRCA of approximately 12 years (95% HPD: 8.6–21.9), indicating a shared ancestry with a Brazilian isolate sampled in 2013. Together, these sequences trace back to a common ancestor dated to around 1951, consistent with the introduction of the Mass vaccine strain [30]. GI-1 was the first IBV lineage described in the United States in the 1930s and later became established across South America. The continued evolution and recombination of GI-1 in Chile, particularly following the 2011 introduction of the 4/91 vaccine, suggest that vaccine pressure may have contributed to the emergence of recombinant variants from 2016 onward.

## 4. Conclusions

This study provides evidence that the use of live-attenuated vaccines with heterologous strains of IBV may lead to the escape of recombinant strains and may replace field strains, causing clinical outbreaks in poultry farms in Chile. We showed that at least four different lineages have been present, circulating in poultry and causing outbreaks with clinical signs in Chile. A historical lineage circulating during the 1980s in Chile, proposed here as GI-31, suggests signatures of inter-lineage recombination with GI-1 vaccine strains and a GI-11-like strain. Then, the GI-16 lineage was detected in isolates associated with clinical outbreaks in flocks in 2009, introduced from China in a similar year to other South American countries. In 2015, four years after the 4/91 vaccine introduction, an isolate suggested a signature of recombination of GI-16 with GI-13. By 2017, all field samples isolated from outbreaks with clinical signs were GI-13, likely derived from the intense use of the 4/91 vaccine. Finally, the current clinical outbreaks in farms in Chile in 2021 have been likely caused by potential recombinant strains derived from two vaccines, GI-1 derived from Mass vaccine strains and GI-13 derived from the 4/91 vaccine. Further genomic sequencing would allow the mapping and identification of specific recombination breakpoints along the viral genome required to directly assess the role of vaccines in shaping the evolution of local IBV strains. Our research extends the understanding of IBV circulating in Chile, encompassing both current and historical viruses, and reveals the potential for exchange of viral segments between the 4/91 vaccine and field strains. This suggests that immunization schedules using live-attenuated vaccines, as employed in several other countries, may alter the genetic diversity of the circulating viral population, potentially suppressing specific strains or recombining with wild types, having implications for the management of this disease.

## Figures and Tables

**Table 1:** Chilean IBV samples used in this study.

ID	Isolation Year	Lineages (S1/N)	Genbank Accession Number (S1/N)
19R6AU-CL-86	1986	GI-31/GI-1	KY861934/MG652290
817AU-CL-86	1986	GI-31/GI-1	KY861933/MG652291
M3010AU-CL-86	1986	GI-31/GI-11	KY861935/MG652289
V819AU-CL-88	1988	GI-31/GI-1	KY861932/MG652292
12124TR-09	2009	GI-16/G1-16	KY861930/MG652294
12101SP-09	2009	GI-16/G1-16	KY861931/MG652293
12101TR-09	2009	GI-16/G1-16	KY861929/MG652295
13347SP-15	2015	GI-16/G1-13	KY861928/MG652296
13691TR-17	2017	GI-13/G1-13	MG545597/MG652300
13698SP-17	2017	GI-13/G1-13	MG545598/MG652299
13861R-17	2017	GI-13/G1-13	MG545599/MG652298
13885R-17	2017	GI-13/G1-13	MG545600/MG652297
15024SP-21	2021	GI-1/G1-13	OQ730098/OQ730093
15011SP-21	2021	GI-1/G1-13	OQ744449/OQ730094
15126SP-21	2021	GI-1/G1-13	OQ730101/OQ730097
15072SP-21	2021	GI-1/G1-13	OQ730100/OQ730096
15060SP-21	2021	GI-1/G1-13	OQ730099/OQ730095

## Data Availability

The datasets generated and/or analyzed in the current study are available in the National Center for Biotechnology Information repository at https://www.ncbi.nlm.nih.gov/. The accession numbers are listed in Table 1.

## Supplementary Materials

The following supporting information can be downloaded at https://www.mdpi.com/article/10.3390/v17081111/s1. Table S1: Estimates of genetic diversity (d) and standard error estimates (SEs) of nucleotide sequences of the S1 gene from IBV lineages, including Chilean samples; Table S2: Estimates of genetic diversity (d) and standard error (SE) estimates of amino acid sequences of the S1 gene from IBV lineages, including Chilean samples. Table S3: Estimates of evolutionary divergence (below diagonal) and standard error estimates (above diagonal) among S1 nucleotide sequences of IBV lineages, including Chilean samples. Table S4: Accession numbers of sequences used in S1 phylogenetic analysis.
